# Supplementary material for: SEGS-1 episomes generated during cassava mosaic disease enhance disease severity
Source: Front Plant Sci. 2025 Jan 10;15:1469045. doi: 10.3389/fpls.2024.1469045 (PMC11795209; doi:10.3389/fpls.2024.1469045)
Supplement: Supplementary Figure 1 — SEGS-1 fragments. (Top) Properties of SEGS-1 fragments. In the primer sequences, black marks SEGS-1 sequences, red marks the NotI linker, and blue marks added residues not in the linker or SEGS-1. (Bottom) The sequences of the SEGS-1 fragments. The green shading indicates the GC-rich region. The blue line marks the junction position. The underlined nucleotides overlap the adjacent fragment. [file DataSheet1.pdf]

Supplementary Table 1 Primers for viral qPCR, SEGS-1 episomes detection, and site-directed mutagenesis

Primer pairs	Sequence (5'-3')	Annealing (°C)	Use
P3P-AA2F†	TCTGCAATCCAGGACCTACC	60	ACMV-DNA qPCR
P3P-AA2R+4R†	GGCTCGCTTCTTGAATTGTC		
EACMVQ1†	GTACCATGCGTCGTTTGAATA	60	EACMV DNA qPCR
EACMVQ2†	GCAAGTCCCAGAGGAAATAGA		
S1-4F*	GGGTAGCCTCTAATCCTTCA	60	End point PCR for SEGS-1 episome detection
S1-2R*	CAGTTGAACTGCTGAACTGC		
SEGS1Nxfwd	CAGCCATCATCGACATCGT	65	*Kpn*1 deletion from pNSB1829
SEGS1Nxrev	AGCAGCCAGTTAGGAAGTTATC		

†Aimone et al., 2022
*Ndunguru et al., 2016

Supplementary Table 2 ANOVA of the effects of SEGS-1 and CMB treatment on log_{10} viral DNA copy number in CMD2 and CMD1 cassava cultivars

**A**	SEGS-1	# Obs	Mean log_{10} copy #	Standard Error	df	F statistic	P value
TME14 (CMD2)	NO	100	3.60	0.098	1,196	7.38	0.0056
	YES	100	3.99	0.112			
TME3 (CMD2)	NO	38	2.71	0.129	1,72	47.82	<0.0001
	YES	38	4.51	0.233			
TMS30572 (CMD1)	NO	12	2.16	0.493	1,21	1.43	0.245
	YES	12	1.45	0.395			

**B**	Virus treatment	# Obs	Mean log_{10} copy #	Standard Error	df	F statistic	P value
TME14 (CMD2)	ACMV	100	4.13	0.110	1,196	21.5	<0.0001
	EACMCV	100	3.47	0.094			
TME3 (CMD2)	ACMV	38	3.68	0.257	1,72	0.24	0.622
	EACMCV	38	3.55	0.220			
TMS30572 (CMD1)	ACMV	12	2.18	0.484	1,21	1.27	0.272
	EACMCV	12	1.43	0.403			

**C**	Virus treatment	SEGS-1	# Obs	Mean log_{10} copy #	Standard Error	Mean separation α=0.05*
TME14 (CMD2)	ACMV	NO	50	3.90	0.142	A
		YES	50	4.36	0.164	A
	EACMCV	NO	50	3.30	0.124	B
		YES	50	3.63	0.138	B
TME3 (CMD2)	ACMV	NO	19	2.89	0.215	A
		YES	19	4.47	0.396	B
	EACMCV	NO	19	2.54	0.136	A
		YES	19	4.56	0.258	B
TMS30572 (CMD1)	ACMV	NO	6	2.44	0.774	A
		YES	6	1.92	0.635	A
	EACMCV	NO	6	1.87	0.662	A
		YES	6	0.984	0.446	A

*Tukey HSD test

The log_{10} DNA copy numbers of ACMV-A and EACMCV-A were compared within each cultivar. In **A**, the ACMV and EACMCV datasets were combined to test the effect of the absence (NO) or presence (YES) of SEGS-1 on viral infection. In **B**, the absence and presence of SEGS-1 datasets were combined to test the effect of CMB species on infection. In **C**, the mean separations of log_{10} viral DNA copy number are shown for each CMB and SEGS-1 treatment of log_{10} viral DNA copy number.

Supplementary Table 3 Primers for cloning and sequencing cassava genomic DNA

Primer name	Sequence (5'-3')	Annealing (°C)	Product (bp)	
Amplification and cloning of SEGS-1 and flanking genomic regions				
S1_PyF9	TGCGTAGTTTGGTGTGTTACT	55	1639	End point PCR
S1_PyR3	AAATCGCAAGGCTAATGAATCC			
Cloning analysis primers for SEGS-1 (NEB #E1202S)				
S1512A	ACCTGCCAACCAAAGCGAGAAC	55	>1639	Colony PCR and Sanger sequencing
S1513A	TCAGGGTTATTGTCTCATGAGCG			
Overlapping sequencing primers for SEGS-1				
S1-hp1F*	TACGCAGCAGCCATCATCGACATC			
S1-2F *	GCAGTTCAGCAGTTCAACTG			
S1-2R*	CAGTTGAACTGCTGAACTGC			
SII 3F*	AGGACCTTTGGAGCTCGA			
S1-4F*	GGGTAGCCTCTAATCCTTCA			
SII B5R	AAGCTTTACAAAGCTCAGCTTGGA			
SII 6F*	GATAACTTCCTAACTGGCTGC			
S1-6R*	GCAGCCAGTTAGGAAGTTATC			
S1_PyR4	AAATTGGAACCACCACTCCA			
Amplification and sequencing of CMD2-associated mutations†				
G680V-F	ACTATTAGCTGCTCGAAGAAGAG	55	371	G680V & A684G
G680V-R	ATCCACTTGATGGCGTATAACT			
CMD2snEF	AATGCAGAAACTAGGAGGCGGCTT	55	575	V528L
CMD2snER	GGTTCTTCTGTTTAGCCTTCCT			
V528L Seq2	TGAAATGTAGTGAGTCTGCTACCTGT			V528L sequencing

*Primers reported in Ndunguru et al., 2016, *J. Virol.* 90, 4160-4173

†Lim et al., 2022, *Nat. Comm.* 13, 3933

Clone name	SEGS-1 fragment	SEGS-1 coordinates	Primer name	Primer sequence	Annealing (°C)
pNSB2163	G	1–277	SEG1FWDNot1	GAATGCGGCCGCCACTACGCTACGCAGCAGCC	63
			S1GnnRNot1	GAATGCGGCCGCAGAGGAGCGAGGTGGGTG	
pNSB2166	H	275–646	SATII FwdNot1	GAATGCGGCCGCGCAGTTCAGCAGTTCAACTG	48
			SATII RNot1	GAATGCGGCCGCGCTTTTATTAAAAAATTTTGG	
pNSB2165	J	644–756	S1InnFwdNot1	GAATGCGGCCGCTTTACAAAGCTCAGCTTGG	55
			SATII 1RevNot1	GAATGCGGCCGCTGAAGGATTAGAGGCTACCC	
pNSB2164	F	757–1007	S1FnnFwdNot1	GAATGCGGCCGCACTCTATTTTTCCGTTTGG	52
			SEGS1REVNot1	GAATGCGGCCGCGTACCACTACGCTACGCAG	
pNSB2184	N	757–1007 + 1–277	SEGS1NIFwd	GAATGCGGCCGC ACTCTATTTTTCCGTTTGG	58
			SEGS1NrRev	GAATGCGGCCGCAGAGGAGCGAGGTGGGTG	

SEGS-1 Fragments

G

ACGCTACGCAG|CAGCCATCATCGACATCGTATTTTAA*CCAGAGGACCCGTCGACCGCCTGAGCAGCAGCACGTC*
GCACCAGCACCACCGCCGCATCGCGCGCCTGTGAGCCGCCGCACCACTGGATCTCGTGCTCGTGAGCCGCCG
CACGCCGCAACTCTTCATCTACCGCTCGTTTACAGCCCACCTCTGTATCACGCGATTGTGAGCCGCCGACTGCCC
GCCGCACGCCCGCACCTCTGCATCAACTGCTCGTTTGCCACCCACCTCGCTCCTCT

J

*CTGC*AGTTCAGCAGTTCAACTGTAAGCATTTTTTCGTTAAATCTGAAGAAAATAGTTCTGGATAGAATTTTGATTGGT
AAGCATTATGAATTTATTATGACATTCAAGTTTATAGGCATCATAGTGTTGCTTAGGACATACTTAGCTTGTAGTTCCA
GAAAATAGAGTCATTTCTGGTTTTCTTTTACAATGGAGGTGTTTATTCCATTGTAATTTTGAGCTGAGCTTTGTTAAG
GACCTTTGGAGCTCGAGCTTTGTTTACAAGGCATCTTGATAGAGCTTTTCGAGCTCGAATTAGAATTAGGCTCATG
GTTATACTAAAGGGAGTTTTTCATGAGTTTGAGTGCTTCCAAAATTTTTTAATAAAAGC

H

GCTTTACAAAGCTCAGCTTGGATCGATTACACCTCTACTGACCCTACTCAGTTTGGGACTCTGGCTGGGGCCATT
CTCAAAAGCCATTTATCTGGGTAGCCTCTAATCCTTCA

F

ACTCTATTTTTCCGTTTGGTTCTGAGAGAGTACTAAAAAGGAAATCCAACCATATATGATCAAATCTAATGATATAGCT
GGTGAGTACTGCAACATAATTGCAATTTATGCAGTTATTTCTCTTGAATTTGGTATCTGCAATTTATGTATAAATCCCT
AGCAGAATATTTTACTGGAGTGGTGAATATGTGTAGGCTTCACTATGGTGGAAATGGAAATTTGTGTGTGATAACTT
CCTAACTGGCTGCT|GC

N

ACTCTATTTTTCCGTTTGGTTCTGAGAGAGTACTAAAAAGGAAATCCAACCATATATGATCAAATCTAATGATATAGCT
GGTGAGTACTGCAACATAATTGCAATTTATGCAGTTATTTCTCTTGAATTTGGTATCTGCAATTTATGTATAAATCCCT
AGCAGAATATTTTACTGGAGTGGTGAATATGTGTAGGCTTCACTATGGTGGAAATGGAAATTTGTGTGTGATAACTT
CCTAACTGGCTGCT|CAGCCATCATCGACATCGTATTTTAA*CCAGAGGACCCGTCGACCGCCTGAGCAGCAGCAC*
GTCGCACCAGCACCACCGCCGCATCGCGCGCCTGTGAGCCGCCGCACCACTGGATCTCGTGCTCGTGAGCCG
CCGCACGCCGCAACTCTTCATCTACCGCTCGTTTACAGCCCACCTCTGTATCACGCGATTGTGAGCCGCCGACTG
CCCGCCGCACGCCCGCACCTCTGCATCAACTGCTCGTTTGCCACCCACCTCGCTCCTCT

Supplementary Figure 1 SEGS-1 fragments**. (Top)** Properties of SEGS-1 fragments. In the primer sequences, black marks SEGS-1 sequences, red marks the *Not*I linker, and blue marks added residues not in the linker or SEGS-1. **(Bottom)** The sequences of the SEGS-1 fragments. The green shading indicates the GC-rich region. The blue line marks the junction position. The underlined nucleotides overlap the adjacent fragment.

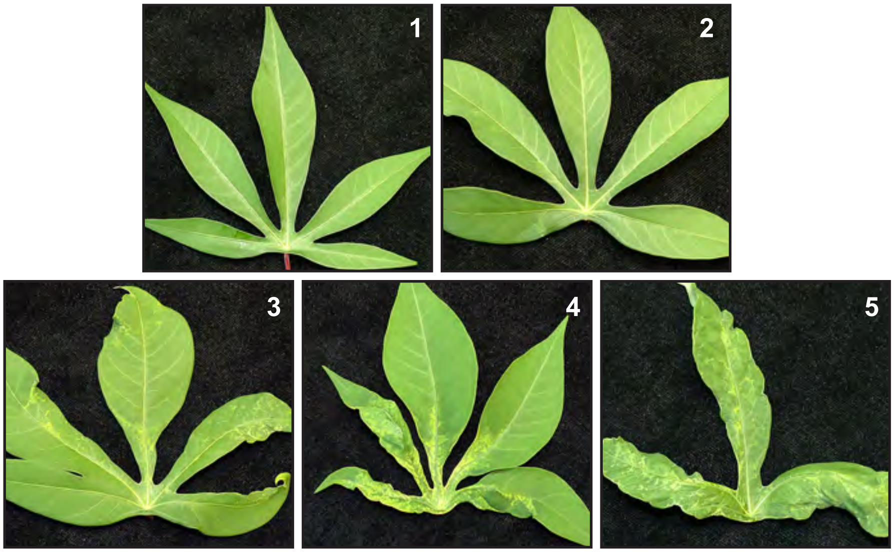

Supplementary Figure 2 CMD symptom scoring. Images of leaves illustrating the symptom scores. A description of the extent of symptoms is below each image.

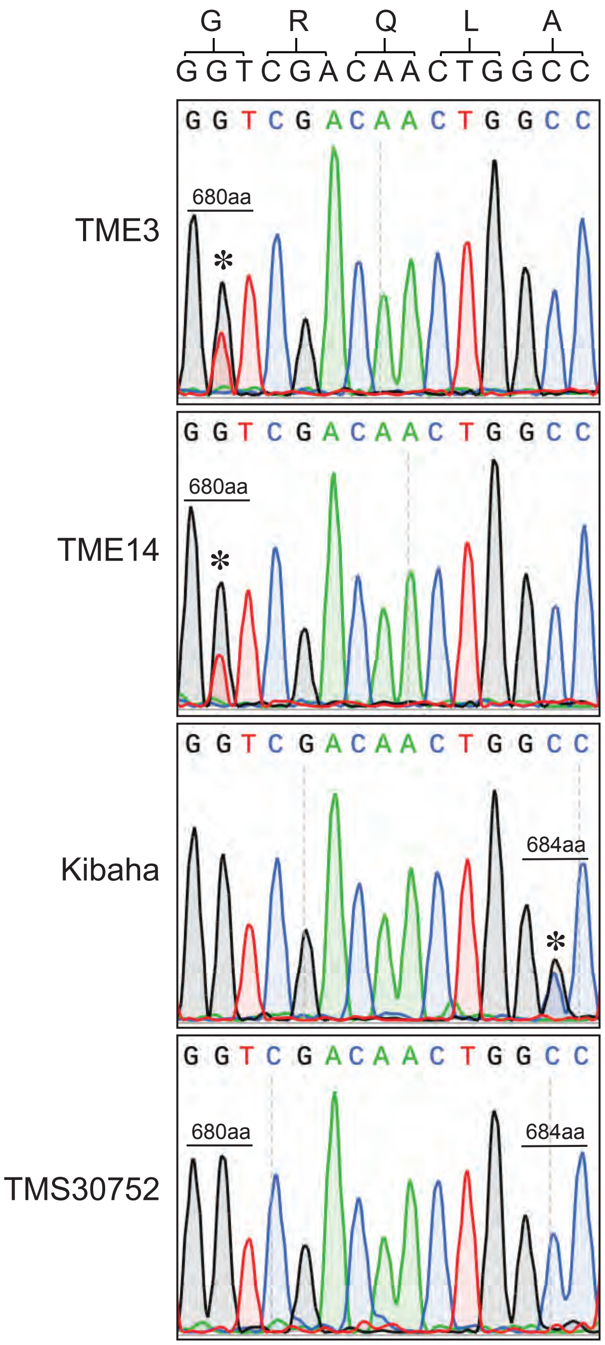

Supplementary Figure 3 Confirmation of CMD2 mutations. Sanger sequencing profiles for the TME3, TME14, Kibaha, and TMS30752 plants used in our studies. The codons in exon 18 of the MePOLD1 gene (Manes. 12G077400) containing the nonsynonymous SNP haplotypes, G680V and A684G, are shown at the top. The G680V and A684G SNPs are highlighted in red in each profile and indicated by asterisks (*). The TME3 and TM14 chromatograms show double peaks in codon 680 indicating that they are chimeric for wild-type GGT (glycine) and mutant GTT (valine) at this position. The Kibaha chromatogram shows a double peak in codon 684 indicating that it is chimeric for wild-type GCC (alanine) and mutant GGC (glycine) at this position. TMS30752, which is not a CMD2 cultivar, does not have a mutation at either position.
